# Natriuretic Peptides as Adjunctive Biomarkers for the Detection of Paroxysmal Atrial Fibrillation in Patients With Cryptogenic Stroke: Systematic Review and Meta‐Analysis

**DOI:** 10.1002/clc.70501

**Published:** 2026-11-05

**Authors:** Mauricio Manrique‐Samer, Santiago Rhenals Acuña, Juan Felipe Betancourt, Oscar Mauricio Muñoz Velandia

**Affiliations:** ^1^ Department of Internal Medicine Pontificia Universidad Javeriana Bogotá Colombia; ^2^ Cardiology and Electrophysiology Unit Hospital Universitario San Ignacio Bogotá Colombia; ^3^ Department of Internal Medicine Hospital Universitario San Ignacio Bogotá Colombia

**Keywords:** atrial fibrillation, brain natriuretic peptide, cryptogenic stroke, ROC curve, telemetry

## Abstract

**Introduction:**

Paroxysmal atrial fibrillation (PAF) is a common cause of cryptogenic stroke (CS). It is often asymptomatic, and prolonged electrocardiographic monitoring is not widely available. Natriuretic peptides (NPs) have emerged as potentially adjunctive biomarkers to support risk stratification.

**Methods:**

A systematic search was conducted in PubMed, Embase, CENTRAL, Scopus, Web of Science, and LILACS up to June 2025. Studies in adults with CS that measured NT‐proBNP, BNP, or MR‐proANP, and that used electrocardiographic monitoring as a reference standard, were included. Risk of bias was assessed using the QUADAS‐2 tool, and data were meta‐analyzed using a bivariate model and a hierarchical summary receiver operating characteristic model. Certainty of the evidence was assessed in accordance with the GRADE methodology.

**Results:**

Eight observational studies involving 1616 patients were included, with an overall PAF prevalence of 19%. For NT‐proBNP and BNP, respectively, pooled sensitivity was 78% (95% CI, 63%–87%) and 75% (95% CI, 56%–87%); specificity, 64% (95% CI, 53%–73%) and 67% (95% CI, 45%–84%); and diagnostic odds ratio, 6.18 (95% CI, 3.94–9.70) and 6.10 (95% CI, 4.42–8.42). The certainty of the evidence was low for NT‐proBNP and moderate for BNP. Heterogeneity was associated with selected cutoff values, analytical platform, and reference‐standard monitoring.

**Conclusion:**

NPs are potentially useful adjunctive biomarkers to support PAF risk stratification in patients with CS. However, the certainty of evidence for NT‐proBNP was low, and for BNP moderate. Incorporating them into stepwise strategies may help stratify patients, but their incorporation requires prospective validation. NPs alone should neither guide anticoagulation nor replace rhythm monitoring.

## Graphical abstract

NT‐proBNP and BNP are accessible biomarkers of atrial stress that show moderate accuracy for detecting paroxysmal atrial fibrillation after cryptogenic stroke. Used as adjunctive biomarkers, they may support stepwise risk stratification and help guide the selective use of prolonged ECG monitoring.

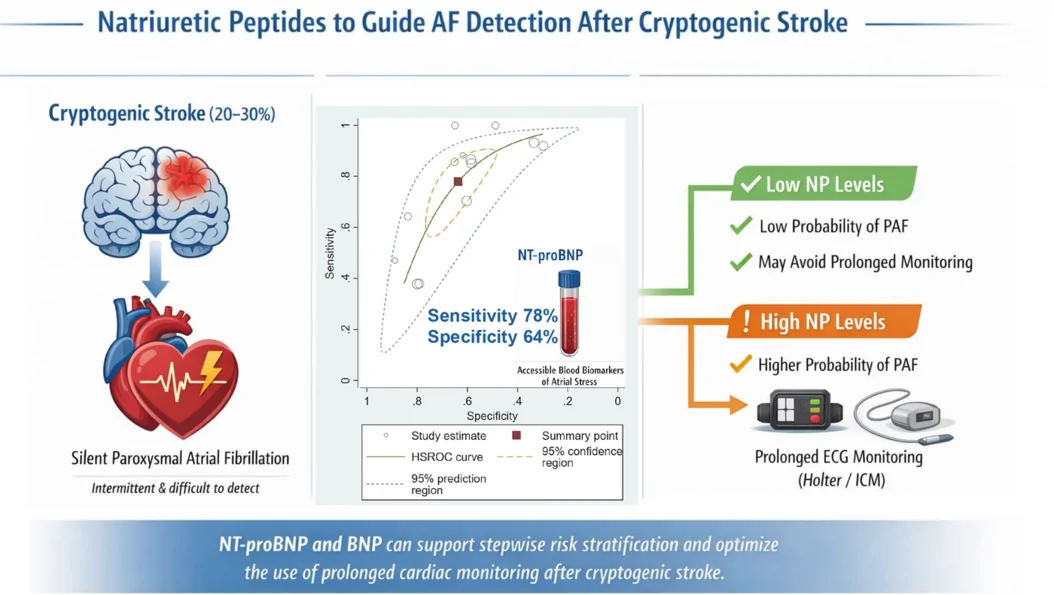

**Abbreviations**
AF: atrial fibrillation
AUC: area under the curve
BNP: B‐type natriuretic peptide
CS: cryptogenic stroke
DOR: diagnostic odds ratio
ECG: electrocardiogram
ESUS: embolic stroke of undetermined source
HSROC: hierarchical summary receiver operating characteristic
ICM: implantable cardiac monitor
LA: left atrium
MR‐proANP: mid‐regional atrial natriuretic peptide
NPs: natriuretic peptides
NT‐proBNP: N‐terminal pro B‐type natriuretic peptide
PAF: paroxysmal atrial fibrillation
ROC: receiver operating characteristic
SROC: summary receiver operating characteristic
TOAST: Trial of Org 10172 in Acute Stroke Treatment

## 1 Introduction

Stroke is the second most common cause of death from cardiovascular disease, and one of the leading causes of morbidity worldwide [1]. Ischemic stroke is considerably more prevalent than hemorrhagic stroke, accounting for 87% of cases [2], and is associated with traditional modifiable risk factors, such as hypertension, diabetes mellitus, dyslipidemia, and smoking, as well as with nonmodifiable factors, such as advanced age, race or ethnicity, and genetics [3]. According to the TOAST classification, the etiologies of ischemic stroke are grouped into large vessel atherosclerosis, cardioembolism, small vessel disease, and other determined or undetermined etiologies [4]. The latter group, known as cryptogenic stroke (CS), represents a diagnostic challenge and carries a high burden for healthcare systems. This has led to a special interest in investigating this group in recent decades [5].

CS accounts for 20%–30% of all ischemic strokes [6, 7]. It is defined according to the TOAST criteria as an ischemic event confirmed by imaging, histopathological findings, or autopsy data, in which no source attributable to cardioembolism, large vessel atherosclerosis, or small vessel disease can be identified despite a thorough evaluation including imaging, cardiovascular, hematological, and serological assessments [4, 6]. In this context, silent paroxysmal atrial fibrillation (PAF) is the most frequently identified underlying etiology [8, 9], and it is considered the main risk factor for cardioembolic stroke [10], increasing the risk of stroke by up to fivefold, particularly in older adults [11]. However, its detection is particularly complex due to the intermittent and frequently asymptomatic nature, which limits its identification by conventional methods.

The detection of silent PAF faces significant challenges. The 12‐lead electrocardiogram (ECG), although highly specific, has limited sensitivity for detecting paroxysmal episodes [12, 13]. Short‐term ambulatory monitoring, such as 24‐ to 72‐h Holter monitoring improves this sensitivity, but is often insufficient in many cases [14, 15]. Extended monitoring strategies, such as event recorders or external loop monitors, offer greater diagnostic yield [15], though their effectiveness relies on patient adherence. Implantable cardiac monitors (ICMs) provide continuous monitoring with high sensitivity [16], but imply invasive procedures and are associated with substantial costs [17]. Emerging tools, such as smart wearable devices and artificial intelligence‐based analysis of cardiac signals, show promising diagnostic potential; however, their usefulness is still under investigation [18, 19].

Given these limitations, natriuretic peptides (NPs), including B‐type natriuretic peptide (BNP), its N‐terminal inactive fragment (NT‐proBNP), and mid‐regional A‐type natriuretic peptide (MR‐proANP), have emerged as promising biomarkers with potential utility as adjunctive biomarkers to identify patients who may benefit from further investigation for silent PAF [20, 21, 22], as they are secreted in response to atrial stress [23, 24, 25]. Several studies evaluating their diagnostic performance in patients with CS have demonstrated a high negative predictive value (NPV) for the detection of PAF [26, 27, 28, 29], supporting their potential utility as an accessible and low‐cost tool to identify patients with a low probability of having PAF.

Although two previous meta‐analyses evaluating the diagnostic accuracy of NPs for detecting PAF in CS have been published [21, 29], both present important methodological limitations. Notably, marked clinical heterogeneity was observed, partly attributable to the inclusion of studies using inconsistent definitions of CS, often including patients who did not strictly meet the diagnostic criteria for this entity (e.g., patients without a complete etiologic evaluation). Moreover, neither conducted a formal risk of bias assessment of the included studies. In addition, one of them did not report estimates of sensitivity or specificity, thereby limiting the applicability of its findings to clinical practice.

This systematic review and meta‐analysis aims to rigorously evaluate the current evidence regarding the use of NPs as adjunctive biomarkers to support the detection of PAF in patients with CS.

## 2 Methods

The protocol for this systematic literature review and meta‐analysis was previously registered and published at PROSPERO [30] (CRD420251086324). The research protocol was reviewed and approved by the Institutional Ethics Committee of the Pontificia Universidad Javeriana‐Hospital Universitario San Ignacio. The objective of the review was to determine whether NPs could identify patients with CS who may benefit from additional testing to confirm PAF. NP thresholds were evaluated against accepted electrocardiographic monitoring reference standards, including 24‐ to 72‐h Holter monitoring, external monitors, and ICMs, using sensitivity, specificity, and false‐positive and false‐negative rates. To conduct this systematic review, we followed the reporting guidelines outlined in PRISMA‐DTA 2020 (Preferred Reporting Items for Systematic Reviews and Meta‐Analyses of diagnostic test accuracy studies) [31].

### 2.1 Eligibility Criteria and Sources of Information

Eligible studies included patients with CS defined according to the TOAST criteria [4]. In addition, studies had to evaluate the diagnostic performance of at least one NPs (BNP, NT‐proBNP, or MR‐proANP) measured using any commercial platform. Moreover, they had to compare them with at least one of the accepted electrocardiographic monitoring reference standards for the diagnosis of PAF (24‐ to 72 h Holter, event recorder, or ICM). Eligible reports were also required to provide sufficient information to construct a 2 × 2 contingency table for at least one NP threshold. Studies in pregnant women, single‐arm designs, those including patients with heart failure, or conducted in non‐human models were excluded.

The literature search was conducted on July 3, 2025, using the following databases: PubMed, Embase (Ovid), Cochrane CENTRAL, Scopus, LILACS, and Web of Science databases. Studies published in English or Spanish were considered.

### 2.2 Search Strategy

The following terms were used to identify relevant studies, combining MeSH descriptors and free‐text terms: (“Stroke” OR “Ischemic Stroke” OR “Stroke, Embolic” OR “Brain Ischemia” OR “cryptogenic stroke”) AND (“Natriuretic Peptides” OR “Natriuretic Peptide, Brain” OR “NT‐proBNP” OR “BNP” OR “MR‐proANP” OR “atrial natriuretic peptide”) AND (“Electrocardiography, Ambulatory” OR “Telemetry” OR “Event Recorder, Cardiac” OR “Holter” OR “ambulatory ECG” OR “implantable loop recorder” OR “insertable cardiac monitor”) AND (“Sensitivity and Specificity” OR “Predictive Value of Tests” OR “ROC Curve” OR “area under curve” OR “diagnostic accuracy”). Because this review specifically evaluated threshold‐based diagnostic accuracy rather than biomarker association or prognostic prediction, diagnostic‐performance terminology was included in the search strategy. No date restrictions were applied. Full details of the search strategies used for each database are provided in Supporting Information S1: Table 1. Reference lists of relevant systematic reviews and included primary studies were additionally screened for potentially eligible reports.

### 2.3 Study Selection and Data Extraction

The retrieved records were managed using the Rayyan tool [32], where duplicates were automatically removed. The selection process was conducted independently and in a blinded manner by two reviewers (M.M.‐S. and S.R.A.) who screened titles, abstracts, and full texts. Discrepancies were resolved by consensus or through consultation with a third reviewer (O.M.M.V.). A manual review of the reference lists of the selected articles was also conducted.

The full texts of all selected articles were retrieved and independently assessed. The following data were extracted from each study: first author, year of publication, study design, sample size, type of NP measured, analytical platform, timing of NP measurement, including whether sampling occurred within 72 h of stroke onset, participant age and sex, reference‐standard method, and measures of LA size, including diameter or volume. In studies where both BNP and NT‐proBNP were measured within the same cohort, all participants underwent assessment with both biomarkers. For analytical purposes, each biomarker was treated as an independent index test and included only in its respective biomarker‐specific meta‐analysis, thereby avoiding double‐counting of participants within any single pooled estimate.

For each study, we also recorded the cutoff value and the corresponding sensitivity, specificity, false positive (FP) rate, and false negative (FN) rate. When multiple cutoff points were reported, data were collected for each. When relevant data were not fully reported, the corresponding authors of the primary studies were contacted, and data from subanalyses not reported in the original articles were obtained whenever possible.

### 2.4 Assessment of Risk of Bias and Certainty of Evidence

The risk of bias in the included studies was assessed using the QUADAS‐2 (Quality Assessment of Diagnostic Accuracy Studies) tool [33], which evaluates four domains: patient selection, index test, reference standard, and flow and timing of testing. The assessment was conducted in four stages: formulation of the review question, adaptation of the signaling questions, construction of study‐specific flowcharts, and classification of the risk of bias as “low,” “high,” or “unclear.”

In addition, the GRADE (Grading of Recommendations Assessment, Development and Evaluation) approach [34] was used to assess the certainty of the evidence for each outcome. This assessment considered the six domains proposed by this methodology: risk of bias, indirectness, inconsistency, imprecision, publication bias, and, when applicable, factors that could increase certainty.

### 2.5 Data Analysis

Statistical analysis included the estimation of diagnostic accuracy measures. For this purpose, 2 × 2 contingency tables were used to calculate sensitivity and specificity for each study and for each reported cutoff point. For each case, the absolute numbers of true positives (TP), true negatives (TN), FP, and FN were identified. When these values were not directly reported, they were derived from available data, including sensitivity, specificity, NPV, positive predictive value (PPV), prevalence, and sample size. Individual results were graphically represented using forest plots, displaying sensitivity and specificity with their corresponding 95% CI, generated in RevMan 5.4.

For the meta‐analysis of diagnostic test accuracy studies, we considered that both the NP (index test) and the reference test (gold standard) were dichotomous. We used four variables as input within each study: TP, FP, FN, and TN. We fitted a two‐level mixed logistic regression model, with independent binomial distributions for the TP and TN conditional on the sensitivity and specificity in each study, and a bivariate normal model for the logit transforms of sensitivity and specificity between studies. Given their mathematical equivalence in diagnostic test accuracy meta‐analysis, both hierarchical bivariate and hierarchical summary receiver operating characteristic (HSROC) models were fitted. Pooled sensitivity, specificity, and diagnostic odds ratio (DOR) estimates were obtained from the bivariate model, whereas HSROC curves were used to visually summarize diagnostic performance and illustrate confidence and prediction regions. Model estimation was performed using maximum likelihood procedures within the hierarchical framework. Model convergence was checked by inspection of the likelihood profiles and the final mL output, and by ensuring that between‐study variances and correlations were estimable and stable. Summary sensitivity and specificity with their 95% confidence region and 95% prediction region were displayed using the HSROC plot option, which provides a graphical quantification of the uncertainty and between‐study heterogeneity, rather than a single AUC estimate. Analysis was performed in Stata (Statacorp, release 16, College Station, TX, USA) using the metandi extension.

Given the variability in diagnostic thresholds across studies, priority was given to the HSROC curve [35]. Assuming that the model is correct, the 95% prediction region indicates the range in which the sensitivity and specificity of a future study are expected to lie.

Prespecified subgroup analyses were performed to explore the effect of duration of monitoring used as the reference standard (< 72 h, 4 weeks, and ≥ 1 year) and of patient age (≤ 65 vs. > 65 years, < 70 vs. ≥ 70 years, and < 75 vs. ≥ 75 years). The results of these analyses were presented in forest plots with 95% CI and ROC plots.

Although a subgroup analysis based on timing of NPs sampling was initially planned, this was deemed not necessary, as the vast majority of studies performed NPs measurement in the first 72 h after the cerebrovascular event. Likewise, a subgroup analysis by type of diagnostic platform was not conducted, since all studies that reported the analytical platform used Roche Diagnostics, precluding meaningful comparison between platforms.

To evaluate the heterogeneity according to the reference standard, we evaluated the feasibility of conducting a meta‐regression; however, given the limited number of included studies (*n* = 8) and the small number of studies per monitoring category (four Holter, two external 28‐day recorders, and two ICM), we determined that a formal meta‐regression would be statistically underpowered and potentially unreliable. Therefore, heterogeneity was explored through stratified subgroup analyses within the hierarchical framework. In our HSROC/bivariate analysis, between‐study heterogeneity was quantified using the between‐study variances of the logit sensitivity and specificity *τ* ^2^_sens_ and *τ* ^2^_spec_. We compared three subgroups: 24–72 h Holter monitoring, 28‐day external recorders, and ICM with ≥ 1 year of follow‐up.

Potential publication bias was assessed using Deeks' funnel plot asymmetry test, which regresses the natural logarithms of DOR against their inverse standard errors, weighted by the inverse square root of the effective sample size. A *p *< 0.10 was considered indicative of statistically significant asymmetry. The corresponding funnel plot was generated using Stata (Statacorp, release 16, College Station, TX, USA).

## 3 Results

### 3.1 Study Selection

The systematic search of the six selected databases yielded a total of 253 records. The selection process is summarized in the PRISMA flow diagram (Figure 1). A total of eight studies met the inclusion criteria and were included in the systematic review.

**Figure 1:**
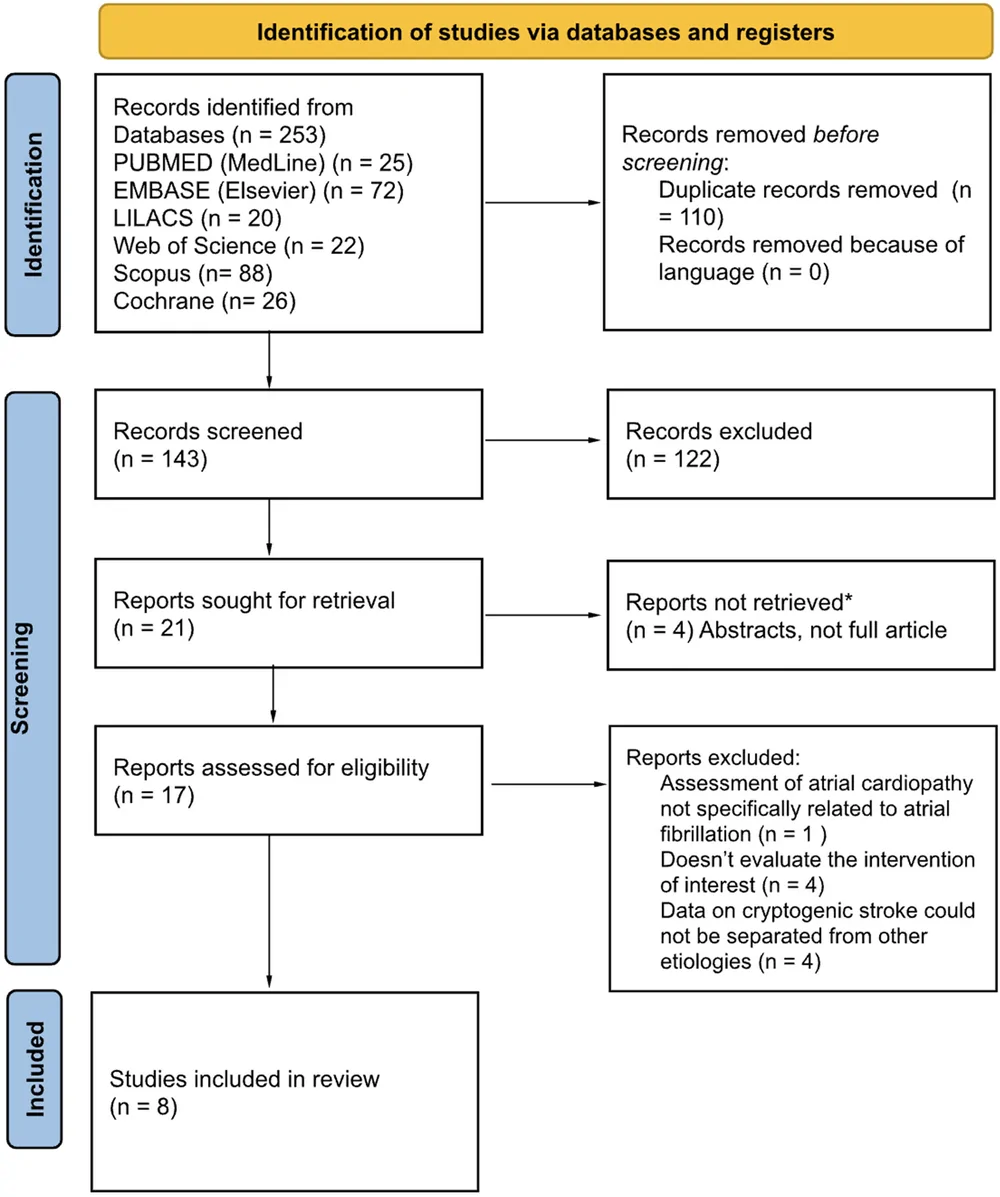
PRISMA flow diagram of study selection.

### 3.2 Characteristics of the Included Studies

The main characteristics of the patients evaluated in each of the included primary studies are summarized in Table 1. In total, data from 1616 patients were analyzed, of whom 1350 underwent NT‐proBNP measurement, and 743 underwent BNP measurement. The median age was 72.5 years, with a slight predominance of male participants (53.7%). The overall prevalence of PAF in the included studies was 19%. All studies employed a prospective observational design. Biomarker measurements were performed within the first 72 h of the index event in all studies except one [36]. In those that reported the analytical platform, Roche Diagnostics was used. As the reference standard, four studies used Holter monitoring ≤ 72 h as reference, two used 4‐week Holter, and two used ICM ≥ 1 year.

**Table 1:** Characteristics of included studies.

Author	Prospective observational	*N* ^a^	Type of NP	Measurement technique	Measurement in first 72 h	Reference standard	Measurement technique	Age	Sex (H)	Measurement of LA
Rodríguez‐Yáñez et al. 2013	Yes	264	NT‐proBNP	Roche Diagnostics (ELECSYS 2010 System)	Yes	24 h Holter	NR	Mean: 70.6 years SD ± 12.1 years	56.9%	NR
Fonseca et al. 2014	Yes	80	NT‐proBNP	Roche Diagnostics (ELECSYS 2010 System)	Yes	24 h Holter on three occasions	NR	Median: 74 years IQR: 7 years	57.2%	LA end‐systolic diameter > 40 mm With AF 58.8% Without AF 33.3%
Kneihsl et al. 2019	Yes	143	NT‐proBNP	Roche Diagnostics (COBAS c8000)	Yes	24 h Holter	NR	Mean: 77.4 ± 9.5 years	49.5%	NR
Pagola et al. 2021	Yes	253	NT‐proBNP	Roche Diagnostics (COBAS c8000)	Yes	Ambulatory Holter monitor for 28 days	Nuubo handheld device	Mean: 74.4 ± 9.1 years	50.4%	With AF: 33.5 mL/m^2^. Without AF: 29 and 31.5 mL/m^2^
Palá et al. 2021	Yes	218	BNP NT‐proBNP	BNP: ADVIA Centaur (Siemens Healthcare) NT‐proBNP: Roche Diagnostics (COBAS c8000)	Yes	Ambulatory Holter monitor for 28 days	Nuubo handheld device	Mean 77.74 ± 7.32 years	50%	NR
Lambert et al. 2023	Yes	259	BNP NT‐proBNP	NR	No	12‐month implantable cardiac monitor	Reveal LINQ (Medtronic)	Mean 65.2 ± 12.6 years	41.7%	NR
Kanyu et al. 2024	Yes	133	NT‐proBNP	NR	Yes	Holter 72 h	NR	Mean 73.08 ± 3.97 years	61.5%	NR
Moriyama et al. 2024	Yes	266	BNP	NR	Yes	Insertable monitor for 1.5 years	Reveal LINQ (Medtronic) Confirm Rx (Abbott) BioMonitor 2‐AF (Biotronik)	Mean 68.1 SD ± 12.0 years	62.8%	Low‐BNP: 33.5 ± 4.35 mm Medium BNP: 34.9 ± 5.5 mm High‐BNP: 36.2 ± 6.54 mm

Abbreviations: AF, atrial fibrillation; BNP, B‐type natriuretic peptide; IQR, interquartile range; LA, left atrium; NP, natriuretic peptide; NR, not reported; NT‐proBNP, N‐terminal B‐type natriuretic peptide; SD, standard deviation.

^a^In studies reporting both BNP and NT‐proBNP, all participants were assessed with both biomarkers. These were analyzed separately within biomarker‐specific meta‐analyses.

### 3.3 Risk of Bias Assessment

The quality assessment of each study, performed using the QUADAS‐2 tool, is shown in Figure 2. Overall, the risk of bias was low in most studies [36, 37, 38, 39]. However, the study by Rodríguez‐Yáñez et al. [28] was judged to have a high risk of bias given that only 24 h Holter monitoring was used as the reference standard, a tool with limited sensitivity for the detection of PAF, and its inconsistent application across participants. In addition, the protocol did not specify whether blinding of the index test results was implemented for the interpretation of the reference standard, nor did it specify the time interval between the index test and the initiation of electrocardiographic monitoring. In the study by Kanyu et al. [40], no prespecified cutoff point for NPs was defined. However, it is important to note that no validated diagnostic thresholds currently exist for the detection of PAF.

**Figure 2:**
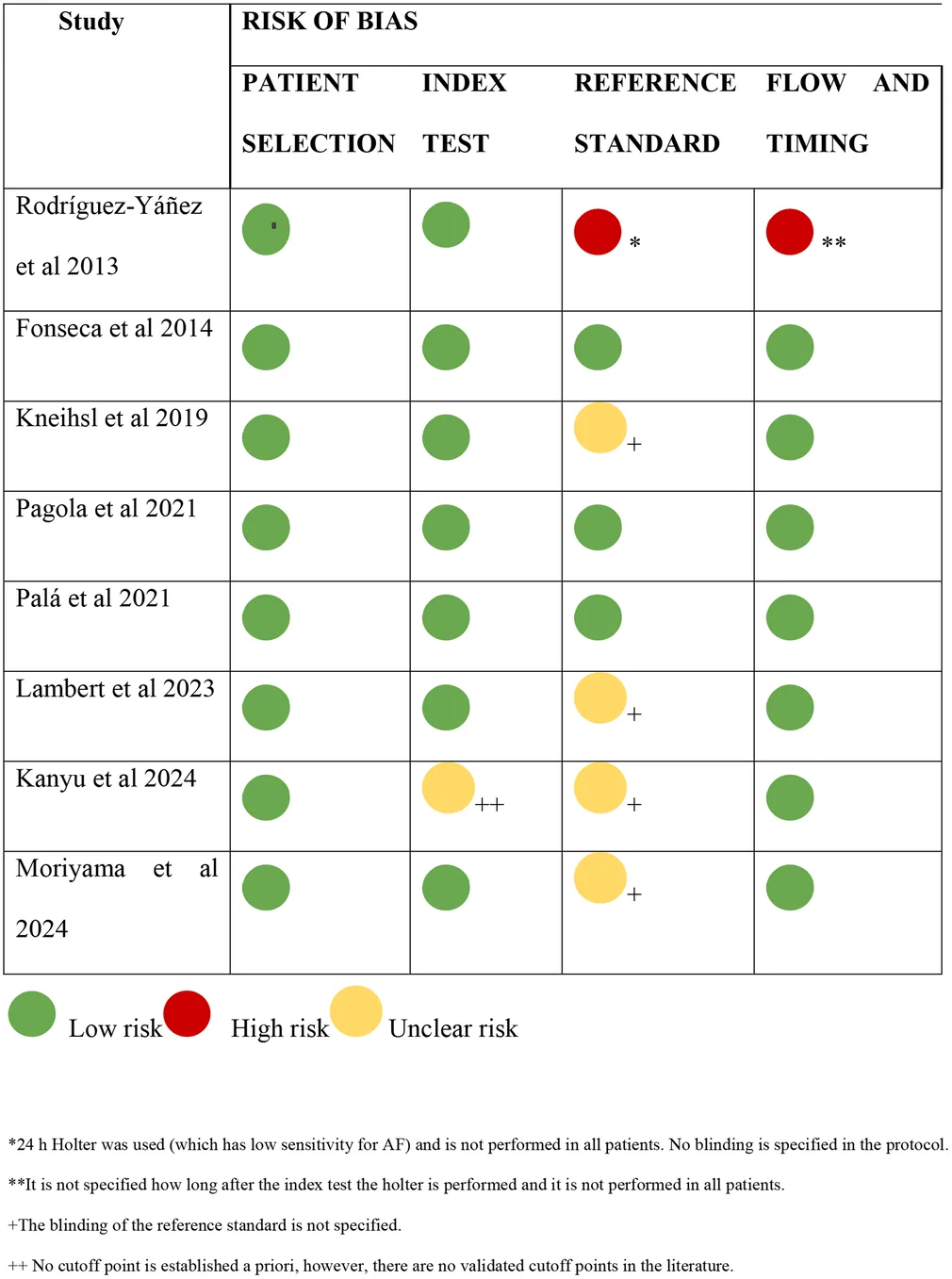
QUADAS 2 tool to assess the methodological quality of diagnostic accuracy studies.

Publication bias was assessed using Deeks’ funnel plot asymmetry test. The test suggested evidence of funnel plot asymmetry (coefficient *b* = 0.94; *z* = 1.77; *p* = 0.076), indicating possible publication bias favoring studies reporting higher DOR (Supporting Information S1: Figure S1). However, this finding should be interpreted cautiously, given the small number of included studies.

A formal sensitivity analysis according with the risk of bias was not performed, as exclusion of the study with the highest risk of bias did not alter the magnitude or direction of the effect.

### 3.4 Diagnostic Performance of Biomarkers

#### 3.4.1 NT‐proBNP

Forest plots showing sensitivity and specificity and constructed in descending order of cutoff point are presented in Figure 3a.

**Figure 3:**
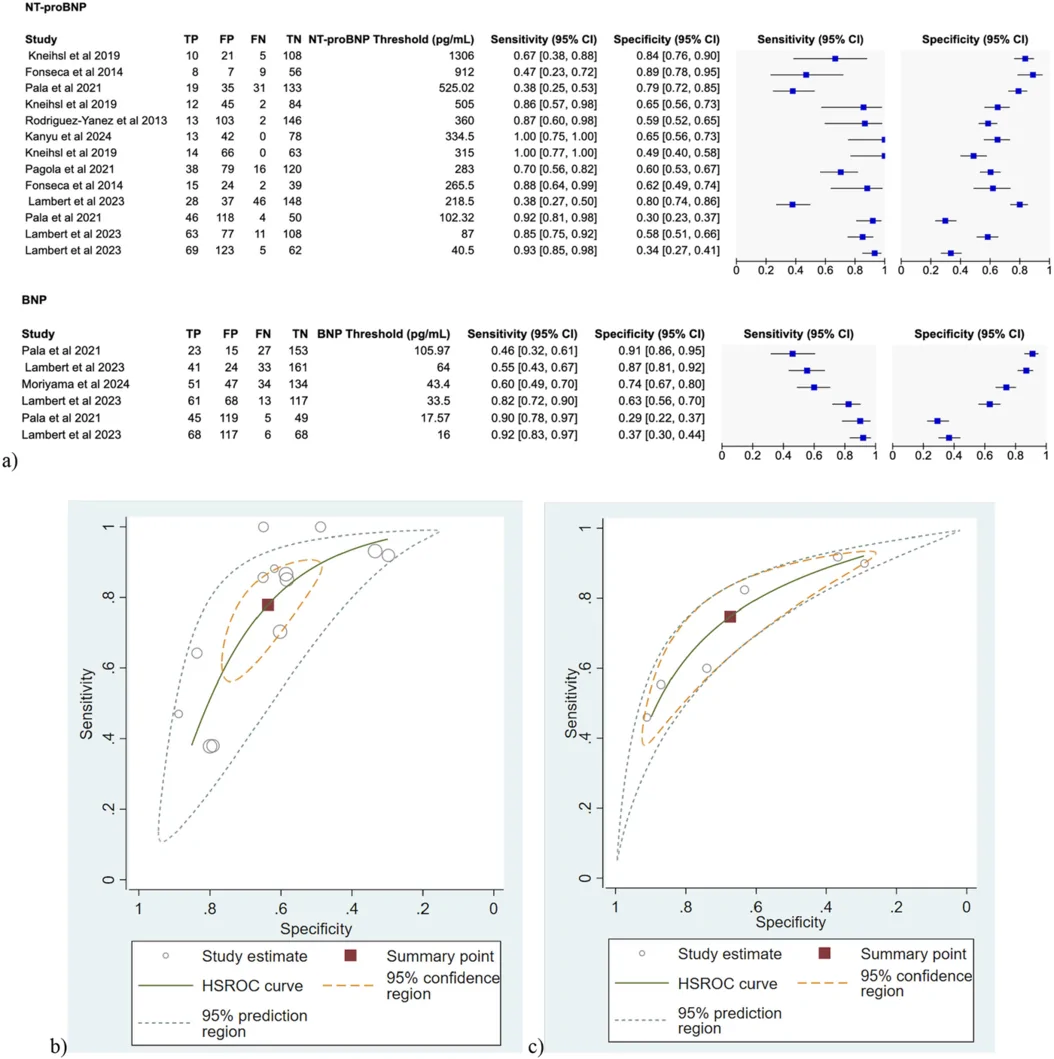
Sensitivity and specificity of natriuretic peptides for screening for paroxysmal atrial fibrillation: (a) Forest plot. (b) Hierarchical SROC curve for NT‐proBNP. (c) Hierarchical SROC curve for BNP.

In addition to the heterogeneity attributable to the cutoff values used, variability was observed related to the measurement platform. A subgroup analysis including only studies that used Roche Diagnostics platforms demonstrated a reduction in heterogeneity (Supporting Information S1: Figure S2). Furthermore, demographic factors such as age may have influenced biomarker levels, as observed in the study by Lambert et al. [36], in which lower cutoff points were applied in a younger cohort.

The HSROC model (Figure 3b) showed an adequate overall discriminative ability of NT‐proBNP for PAF screening. The prediction region reflected variability across studies, probably due to differences in monitoring duration, patient age, and selected threshold. The bivariate model meta‐analysis yielded, for the NT‐proBNP summary point, a sensitivity of 78% (95% CI: 63–87), a specificity 64% (95% CI: 53–73), and a DOR of 6.18 (95% CI: 3.94–9.70).

#### 3.4.2 BNP

Similarly, forest plots stratified by cutoff value were constructed for BNP (Figure 3a). Higher cutoff values were associated with increased specificity at the expense of sensitivity, whereas lower cutoff values improved sensitivity but reduced specificity, indicating that heterogeneity was largely attributable to the selected cutoff.

The HSROC model for BNP (Figure 3c) summarized its diagnostic performance across the included studies. The prediction region was relatively narrow, although this finding should be interpreted cautiously given the limited number of BNP studies. Because the BNP and NT‐proBNP models were based largely on different study populations, any comparison between them is exploratory and descriptive rather than a direct comparative inference.

The bivariate model meta‐analysis yielded, for the BNP summary point, a sensitivity of 75% (95% CI: 56–87), specificity of 67% (95% CI: 45–84), and DOR of 6.10 (95% CI: 4.42–8.42).

#### 3.4.3 MR‐proANP

Despite a thorough search, no suitable studies were found that evaluated the diagnostic usefulness of MR‐proANP for PAF screening in patients with CS.

### 3.5 Subgroup Analysis According to Reference Standard

A subgroup analysis was conducted according to the duration of monitoring used as the reference standard: Holter monitoring lasting ≤ 72 h, 28‐day external monitoring, and ICM monitoring for ≥ 1 year. NT‐proBNP appeared to have more favorable diagnostic performance in studies using short‐duration monitoring as the reference standard than in studies using longer monitoring strategies (Figure 4). It is associated with the fact that longer monitoring increases PAF ascertainment and reduces outcome misclassification, thereby affecting the apparent sensitivity and specificity of NPs. The corresponding BNP analysis showed no clear differences across monitoring categories, although no BNP studies used short‐duration monitoring as the reference standard (Figure 5). These findings should be considered exploratory because few studies contributed to each subgroup.

**Figure 4:**
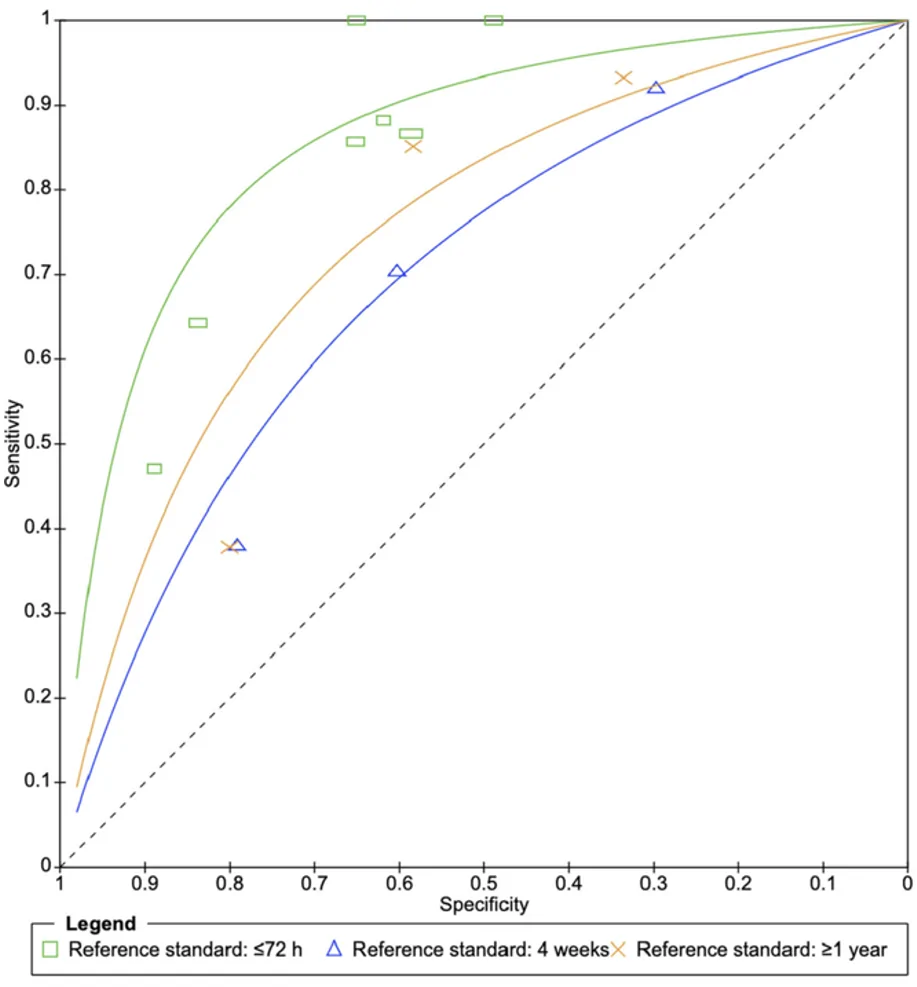
ROC curves for NT‐proBNP according to the reference standard used.

**Figure 5:**
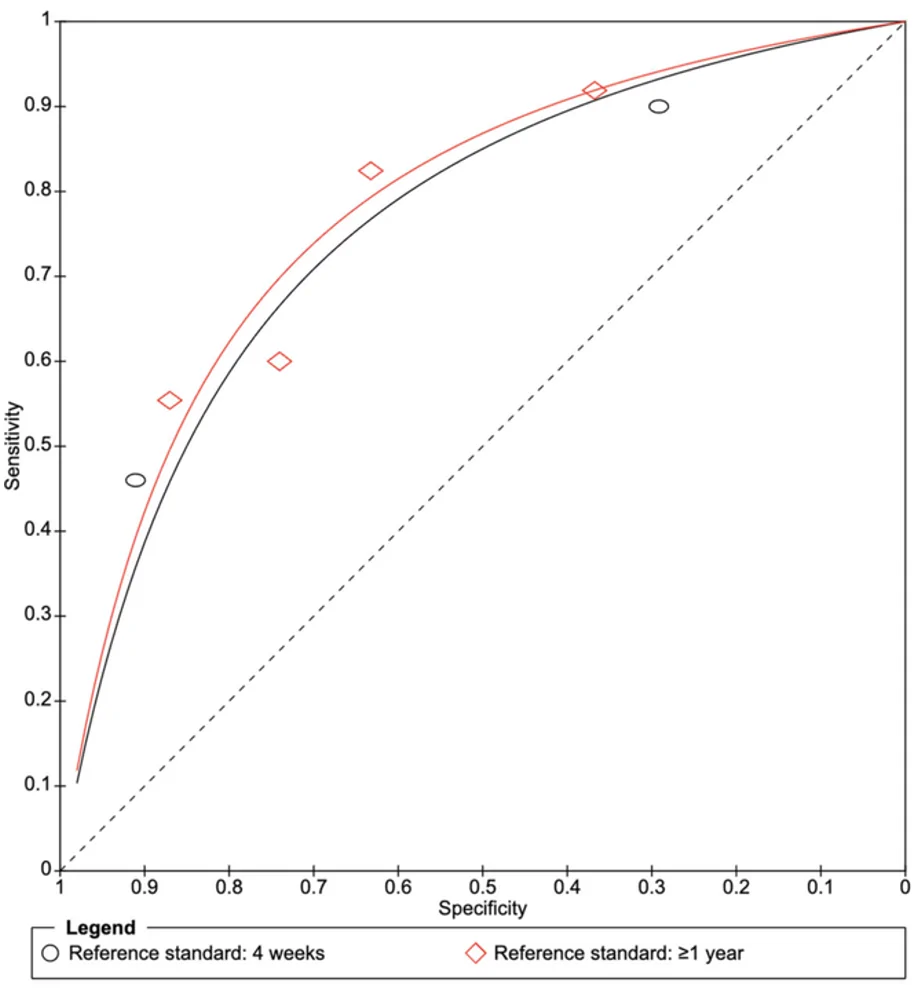
ROC curves for BNP according to the reference standard used.

Heterogeneity evaluation showed that across subgroups, *τ* ^2^_sens_ was relatively similar (≈1.18–1.27), indicating comparable between‐study variability in sensitivity estimates. In contrast, *τ* ^2^_spec_ was higher in the 28‐day recorder subgroup (1.46) than in the 24–72 h Holter and ICM ≥ 1 year subgroups (0.41 and 0.75, respectively), suggesting more pronounced heterogeneity in specificity among studies using 28‐day external recorders. Corresponding between‐study standard deviations (*τ*) ranged from approximately 1.08–1.13 for sensitivity and from 0.64 (24–72 h Holter) to 1.21 (28‐day recorder) for specificity, again highlighting greater variability in specificity in the 28‐day recorder subgroup.

### 3.6 Subgroup Analysis According to Age

A subgroup analysis was also performed according to the mean or median participant age, using thresholds commonly applied in clinical risk scores such as CHA_2_DS_2_‐VASc: ≤ 65 versus > 65 years, < 70 versus ≥ 70 years, and < 75 versus ≥ 75 years [41]. No meaningful differences in NP diagnostic performance were observed across age groups (Supporting Information S1: Figure S3).

### 3.7 Summary of Findings and Certainty of the Evidence Tables

Using pooled sensitivity and specificity from the bivariate model, we generated modeled projections of absolute effects (true/false positives and negatives per 1000) under prevalence scenarios of 10%, 19%, and 30% (Figure 6). These values are not observed counts, but projections derived from pooled estimates, with uncertainty ranges calculated from the 95% CI of sensitivity and specificity. This analysis was conducted using GRADEpro, with 19% as the central prevalence estimate reported in the literature [42, 43].

**Figure 6:**
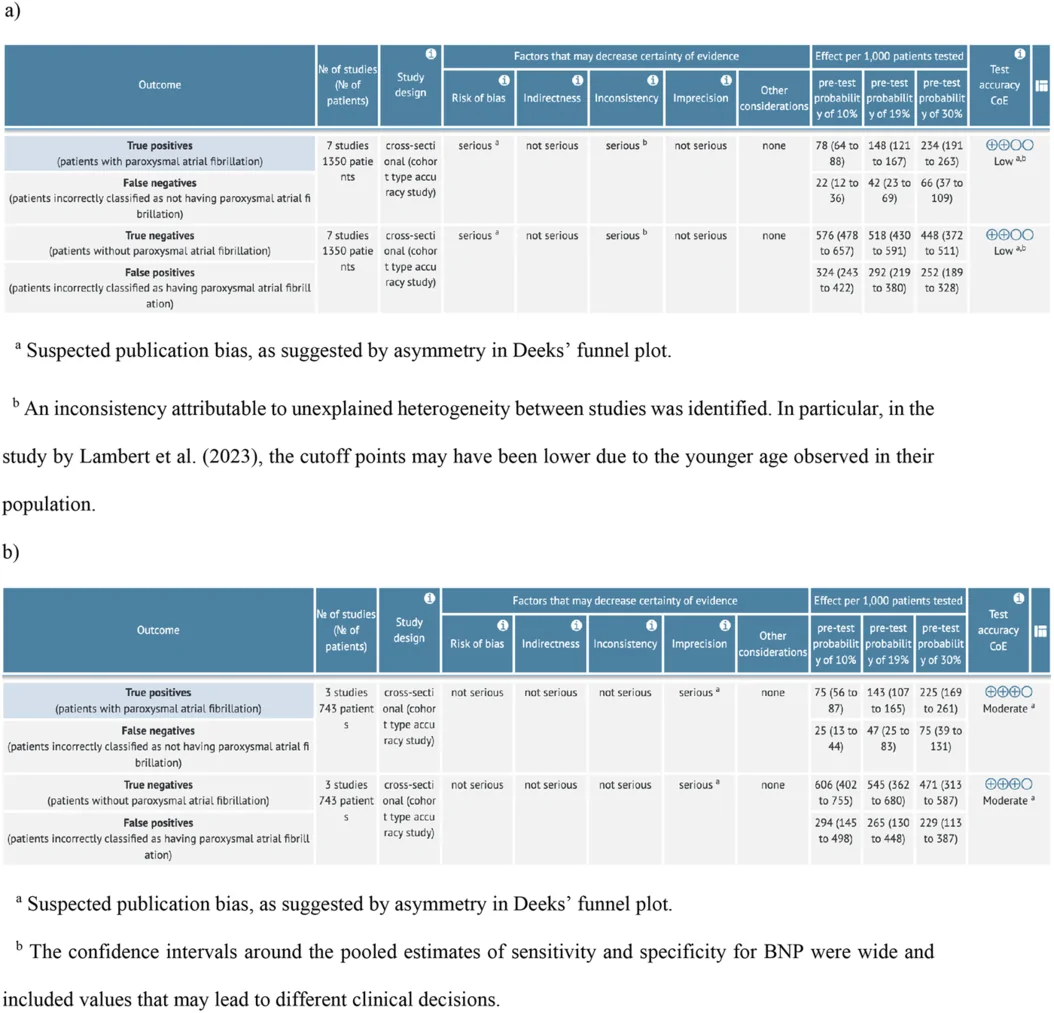
Evidence summary tables: (a) NT‐proBNP; (b) BNP. (a) ^a^Suspected publication bias, as suggested by asymmetry in Deeks’ funnel plot. ^b^An inconsistency attributable to unexplained heterogeneity between studies was identified. In particular, in the study by Lambert et al. [36], the cutoff points may have been lower due to the younger age observed in their population. (b) ^a^Suspected publication bias, as suggested by asymmetry in Deeks’ funnel plot. ^b^The confidence intervals around the pooled estimates of sensitivity and specificity for BNP were wide and included values that may lead to different clinical decisions.

The modeled projections suggest that NT‐proBNP provides a clinically acceptable balance between sensitivity and specificity when used as an adjunctive biomarker strategy for PAF in patients with CS. Specifically, at a prevalence of 19%, the model predicts that the majority of patients with PAF would be identified, with an assumed number of FN (42 per 1000 patients screened), at the cost of a substantial number of FP (292 per 1000 patients screened). The certainty of the evidence for this outcome was rated as low (Figure 6a).

For BNP, the model estimated a slightly higher number of FN (47 patients per 1000 patients screened), and a comparable number of FP (265 per 1000 patients screened). The certainty of the evidence was rated as moderate (Figure 6b).

At a modeled PAF prevalence of 30%, the number of FN increased to 75 per 1000 patients screened, suggesting that NPs should not be used as a rule‐out strategy in higher‐prevalence settings.

## 4 Discussion

The present systematic review evaluated the role of NPs as adjunctive biomarkers to support risk stratification for the detection of PAF in patients with CS. Overall, NT‐proBNP and BNP showed moderate discriminatory capacity; however, these biomarkers should be interpreted as non‐specific markers of atrial stress and structural remodeling that may support further rhythm evaluation, rather than as stand‐alone tests for PAF detection.

It should be emphasized that elevated NT‐proBNP or BNP levels do not imply the direct presence of PAF per se. These biomarkers reflect an atrial wall response to hemodynamic stress and may be elevated due to a variety of causes unrelated to PAF. Therefore, a positive result in patients without heart failure should be interpreted as a marker of atrial dysfunction or stress warranting further complementary diagnostic studies. It is important to consider that a proportion of these cases may correspond to patients with underlying atrial cardiomyopathy in the absence of PAF, a condition that has also been associated with an increased risk of stroke [44] and may justify closer clinical surveillance.

Low NP values, particularly when conservative thresholds are used, exhibit high NPV, which may provide useful information in the risk stratification process. Exploratory inspection of the forest plots suggested NT‐proBNP thresholds around 100 pg/mL and BNP thresholds around 30 pg/mL generally achieved sensitivities exceeding 80%. However, these observations should be prospectively validated. In such cases, low NP values may reduce the post‐test probability of PAF in selected low‐prevalence settings; however, this prevalence‐dependent finding should not be interpreted as a universal rule‐out strategy without prospective validation using prespecified cutoffs.

The reference standards differed substantially in monitoring duration. Longer monitoring increases PAF ascertainment and reduces outcome misclassification, thereby affecting the apparent sensitivity and specificity of NPs. Consequently, the pooled estimates represent an average across heterogeneous monitoring strategies and should not be interpreted as universal estimates applicable to a specific monitoring modality.

The evidence summary tables, developed according to the GRADE methodology, allowed for an evaluation of the clinical implications and practical applicability of implementing a screening strategy for PAF using NP in patients with CS. The analysis suggests that, in settings with an intermediate expected prevalence (19%) of PAF, the strategy would correctly identify 148 patients, while missing 42 patients with PAF, within a population of 1000 patients with clinical suspicion. This level of performance is acceptable from a screening perspective, particularly considering that positive results do not constitute criteria for initiating anticoagulation, but rather act as triggers for confirmatory tests. Especially considering that anticoagulation without strict detection of PAF has been shown to result in net clinical harm [45, 46].

According to the same analysis, this strategy would also yield a relatively high number of FP (between 270 and 290 patients per 1000), meaning that a substantial number of individuals without PAF would still undergo complementary tests. Although these tests would generally not expose patients to inappropriate treatment, they could increase healthcare utilization.

Caution should be exercised when applying an NP‐based strategy in patients with a high pretest probability of PAF, such as those with marked dilated left atrial enlargement, overt atrial dysfunction, or suggestive echocardiographic findings. Although the number of FN appears modest in absolute terms, failure to identify AF after CS has important therapeutic implications because these patients may not receive anticoagulation for secondary prevention. Consequently, NPs should not be considered a rule‐out strategy, and a more intensive diagnostic approach, such as early initiation of extended cardiac monitoring, should be considered.

This systematic literature review overcomes several important limitations present in previously published reviews. Specifically, we restricted the population to a homogeneous operational definition of CS according to the TOAST criteria, provided a formal assessment of the methodological quality of included studies, assessed the certainty of the evidence using the GRADE framework, applied a robust quantitative synthesis approach with hierarchical models, and performed stratified analyses by NPs threshold and relevant subgroup analysis. Furthermore, this review was based on a larger number of studies and evaluated BNP. In contrast to the review performed by Justo et al. [21], we decided not to include the study by Šaňák et al. [47] since the information provided in the article did not allow isolation of data specific to CS patients (81 out of 98 total participants).

Nevertheless, this review has limitations that should be acknowledged. The number of events and the number of studies evaluating each biomarker, particularly BNP, was limited. This limited the precision of the pooled estimates and the assessment of publication bias. Deeks' funnel plot asymmetry test used to evaluate publication bias in this review should be interpreted with caution, and further studies are required to fully assess this potential bias. Additionally, the clinical and methodological heterogeneity observed across the studies could not be fully explained by the assessed subgroup variables. Furthermore, only two studies evaluated BNP and NT‐proBNP within the same cohort. Accordingly, any comparison between their separately pooled estimates is exploratory and descriptive, and no formal head‐to‐head comparative inference can be made. Another limitation was that we had to derive 2 × 2 tables in some cases when the specific data were not reported. While these calculations may introduce minor rounding imprecision, they were based on reported prevalence, sensitivity, specificity, and total sample size, ensuring internal consistency. Given the limited number of studies and the small proportion requiring reconstruction, we considered the potential impact to be minimal.

NPs concentrations are influenced by age, renal function, left atrial size, overt or subclinical structural heart disease, acute stroke severity, and the timing of blood sampling. Although overt heart failure was excluded in most studies and biomarker sampling occurred within 72 h of stroke onset in all but one study, the included reports did not consistently measure, report, or adjust for these determinants. Consequently, residual confounding may have influenced the reported thresholds and diagnostic accuracy estimates. Finally, due to the scope of this review, we were unable to explore the combined performance of multivariable models integrating clinical variables, biomarkers such as NPs, and echocardiographic findings as have emerged in some small studies [48, 49]. Future reviews will be necessary to overcome these difficulties.

## 5 Conclusion

NPs may serve as adjunctive biomarkers for PAF risk stratification in patients with CS, particularly in low‐pretest‐probability settings. However, heterogeneity in monitoring strategies, imprecise estimations, and possible publication bias imply limited certainty of evidence and require cautious interpretation and prospective validation before clinical implementation. NPs alone should neither guide anticoagulation decisions nor replace rhythm monitoring.

## Author Contributions

We hereby affirm that all listed authors have fully satisfied the authorship criteria established by the ICMJE. Each author has made substantial contributions to the conception and design of the study and/or to the acquisition, analysis, and interpretation of data. We have been actively involved in drafting and revising the manuscript for significant intellectual content. We have also provided final approval of the version to be published and accepted responsibility for the accuracy and integrity of the work in its entirety. We have ensured that any concerns regarding its validity are appropriately addressed and resolved.

## Funding

The authors have nothing to report.

## Ethics Statement

The research protocol was reviewed and approved by the Institutional Ethics Committee of the Pontificia Universidad Javeriana‐Hospital Universitario San Ignacio.

## Supporting information


**Figure S1:** Deeks´ funnel plot for assessing publication bias among the included studies for NT‐proBNP.
**Figure S2:** Forest plot of sensitivity and specificity of natriuretic peptides for screening paroxysmal atrial fibrillation stratified by manufacturer's platform.
**Figure S3:** ROC curves for NT‐proBNP and BNP according to age a) ≤65 years. b) ≥70 years. c) ≥75 years.
**Table S1:** Detailed search strategy.
**Table S2:** Definitions and adjudication of atrial fibrillation across included studies.

## Data Availability

The data that support the findings of this study are available in the supplementary material of this article.

## References

[1] GBD 2016 Lifetime Risk of Stroke Collaborators , “Global, Regional, and Country‐Specific Lifetime Risks of Stroke, 1990 and 2016,” New England Journal of Medicine 379, no. 25 (2018): 2429–2437, 10.1056/NEJMoa1804492. PMC6247346 30575491

[2] B. C. V. Campbell , D. A. De Silva , M. R. Macleod , et al., “Ischaemic Stroke,” Nature Reviews Disease Primers 5, no. 1 (2019): 70. 10.1038/s41572-019-0118-8 31601801

[3] T. B. Fandos , “Revisión Actualizada Sobre Enfermedad Cerebrovascular: Estudio De Un Caso,” Nursing (Ed. española) 21, no. 3 (2003): 45–57, 10.1016/S0212-5382(03)71813-5.

[4] H. P. Adams , B. H. Bendixen , L. J. Kappelle , et al., “Classification of Subtype of Acute Ischemic Stroke: Definitions for Use in a Multicenter Clinical Trial. TOAST. Trial of Org 10172 in Acute Stroke Treatment,” Stroke 24, no. 1 (1993): 35–41. 10.1161/01.str.24.1.35 7678184

[5] R. G. Hart , H. C. Diener , S. B. Coutts , et al., “Embolic Strokes of Undetermined Source: The Case for a New Clinical Construct,” Lancet Neurology 13, no. 4 (2014): 429–438, 10.1016/S1474-4422(13)70310-7. 24646875

[6] D. Yang and M. S. V. Elkind , “Current Perspectives on the Clinical Management of Cryptogenic Stroke,” Expert Review of Neurotherapeutics 23, no. 3 (2023): 213–226, 10.1080/14737175.2023.2192403. PMC10166643 36934333

[7] J. L. Saver , “Cryptogenic Stroke,” New England Journal of Medicine 375, no. 11 (2016): e26, 10.1056/NEJMc1609156. 27626542

[8] D. J. Gladstone , M. Spring , P. Dorian , et al., “Atrial Fibrillation in Patients With Cryptogenic Stroke,” New England Journal of Medicine 370, no. 26 (2014): 2467–2477, 10.1056/NEJMoa1311376. 24963566

[9] S. Elsheikh , A. Hill , G. Irving , G. Y. H. Lip , and A. H. Abdul‐Rahim , “Atrial Fibrillation and Stroke: State‐of‐the‐Art and Future Directions,” Current Problems in Cardiology 49, no. 1 pt. C (2024): 102181, 10.1016/j.cpcardiol.2023.102181. 37913929

[10] I. C. Van Gelder , M. Rienstra , K. V. Bunting , et al., “2024 ESC Guidelines for the Management of Atrial Fibrillation Developed in Collaboration With the European Association for Cardio‐Thoracic Surgery (EACTS): Developed by the Task Force for the Management of Atrial Fibrillation of the European Society of Cardiology (ESC), With the Special Contribution of the European Heart Rhythm Association (EHRA) of the ESC. Endorsed by the European Stroke Organisation (ESO),” European Heart Journal 45, no. 36 (2024): 3314–3414, 10.1093/eurheartj/ehae176. 39210723

[11] G. Lippi , F. Sanchis‐Gomar , and G. Cervellin , “Global Epidemiology of Atrial Fibrillation: An Increasing Epidemic and Public Health Challenge,” International Journal of Stroke 16, no. 2 (February 2021): 217–221, 10.1177/1747493019897870. 31955707

[12] D. Ko , M. K. Chung , P. T. Evans , et al., “Atrial Fibrillation: A Review,” JAMA 333, no. 4 (2025): 329–342, 10.1001/jama.2024.22451. PMC11774664 39680399

[13] B. J. J. M. Brundel , X. Ai , M. T. Hills , M. F. Kuipers , G. Y. H. Lip , and N. M. S. de Groot , “Atrial Fibrillation,” Nature Reviews Disease Primers 8, no. 1 (2022): 21, 10.1038/s41572-022-00347-9. 35393446

[14] O. Faust , E. J. Ciaccio , and U. R. Acharya , “A Review of Atrial Fibrillation Detection Methods as a Service,” International Journal of Environmental Research and Public Health 17, no. 9 (2020): 3093, 10.3390/ijerph17093093.

[15] E. Y. Ding , G. M. Marcus , and D. D. McManus , “Emerging Technologies for Identifying Atrial Fibrillation,” Circulation Research 127, no. 1 (2020): 128–142, 10.1161/CIRCRESAHA.119.316342. PMC8386822 32716695

[16] A. Katapadi , N. Chelikam , J. Garg , et al., “Dynamic Data‐Driven Management of Atrial Fibrillation: The MONITOR AF Study,” Heart Rhythm: The Official Journal of the Heart Rhythm Society 22, no. 12 (2025): 3050–3056, 10.1016/j.hrthm.2025.01.011. 39826639

[17] “Electrophysiology Supplies‐Hospital Militar Central [Internet] ,” Colombia Licita, cited July 2, 2025. https://colombialicita.com/licitacion/209207109.

[18] D. Bartusik‐Aebisher , K. Rogóż , and D. Aebisher , “Artificial Intelligence and ECG: A New Frontier in Cardiac Diagnostics and Prevention,” Biomedicines 13, no. 7 (July 2025): 1685, 10.3390/biomedicines13071685. PMC12292989 40722756

[19] P. Karakasis , P. Theofilis , M. Sagris , et al., “Artificial Intelligence in Atrial Fibrillation: From Early Detection to Precision Therapy,” Journal of Clinical Medicine 14, no. 8 (2025): 2627, 10.3390/jcm14082627. PMC12027562 40283456

[20] A. Cameron , H. K. Cheng , R. P. Lee , et al., “Biomarkers for Atrial Fibrillation Detection After Stroke: Systematic Review and Meta‐Analysis,” Neurology 97, no. 18 (2021): e1775–e1789, 10.1212/WNL.0000000000012769. 34504030

[21] A. S. S. Justo , S. M. A. Nóbrega , and A. L. A. Silva , “Cardiac Blood‐Based Biomarkers of Myocardial Stress as Predictors of Atrial Fibrillation Development in Patients With Embolic Stroke of Undetermined Source/Cryptogenic Stroke: A Systematic Review and Meta‐Analysis,” Journal of Clinical Neurology 20, no. 3 (2024): 256–264, 10.3988/jcn.2023.0068. PMC11076184 38171502

[22] B. Musmar , H. A. Salim , B. Mac Grory , et al., “MR‐proANP Levels in Acute Ischemic Stroke and Clinical Outcomes: A Systematic Review and Meta‐Analysis,” Neurosurgical Review 47, no. 1 (2024): 835, 10.1007/s10143-024-03073-w. 39496843

[23] L. B. Daniels , P. Clopton , C. R. deFilippi , et al., “Serial Measurement of N‐Terminal Pro‐B‐Type Natriuretic Peptide and Cardiac Troponin T for Cardiovascular Disease Risk Assessment in the Multi‐Ethnic Study of Atherosclerosis (MESA),” American Heart Journal 170, no. 6 (2015): 1170–1183, 10.1016/j.ahj.2015.09.010. PMC4684596 26678639

[24] S. M. Werhahn , C. Becker , M. Mende , et al., “NT‐proBNP as a Marker for Atrial Fibrillation and Heart Failure in Four Observational Outpatient Trials,” ESC Heart Failure 9, no. 1 (2022): 100–109, 10.1002/ehf2.13703. PMC8788004 34850596

[25] T. Breidthardt , M. Noveanu , S. Cayir , et al., “The Use of B‐Type Natriuretic Peptide in the Management of Patients With Atrial Fibrillation and Dyspnea,” International Journal of Cardiology 136, no. 2 (2009): 193–199, 10.1016/j.ijcard.2008.04.045. 18619686

[26] E. Palà , J. Pagola , J. Juega , et al., “B‐Type Natriuretic Peptide Over N‐Terminal Pro‐Brain Natriuretic Peptide to Predict Incident Atrial Fibrillation After Cryptogenic Stroke,” European Journal of Neurology 28, no. 2 (2021): 540–547, 10.1111/ene.14579. 33043545

[27] M. Kneihsl , T. Gattringer , E. Bisping , et al., “Blood Biomarkers of Heart Failure and Hypercoagulation to Identify Atrial Fibrillation‐Related Stroke,” Stroke 50, no. 8 (2019): 2223–2226, 10.1161/STROKEAHA.119.025339. 31216968

[28] M. Rodríguez‐Yáñez , S. Arias‐Rivas , M. Santamaría‐Cadavid , T. Sobrino , J. Castillo , and M. Blanco , “High Pro‐BNP Levels Predict the Occurrence of Atrial Fibrillation After Cryptogenic Stroke,” Neurology 81, no. 5 (2013): 444–447, 10.1212/WNL.0b013e31829d8773. 23803318

[29] J. Patel and S. M. M. Bhaskar , “Diagnostic Utility of N‐Terminal Pro‐B‐Type Natriuretic Peptide in Identifying Atrial Fibrillation Post‐Cryptogenic Stroke: A Systematic Review and Meta‐Analysis,” Pathophysiology 31, no. 3 (2024): 331–349, 10.3390/pathophysiology31030024. PMC11270372 39051222

[30] “PROSPERO: International Prospective Register of Systematic Reviews [Internet],” Centre for Reviews and Dissemination, University of York. https://www.crd.york.ac.uk/prospero/.

[31] J. P. Salameh , P. M. Bossuyt , T. A. McGrath , et al., “Preferred Reporting Items for Systematic Review and Meta‐Analysis of Diagnostic Test Accuracy Studies (PRISMA‐DTA): Explanation, Elaboration, and Checklist,” BMJ 370 (August 2020): m2632, 10.1136/bmj.m2632. 32816740

[32] M. Ouzzani , H. Hammady , Z. Fedorowicz , and A. Elmagarmid , “Rayyan: A Web and Mobile App for Systematic Reviews [Internet],” Rayyan Systems Inc, cited August 4, 2025. https://new.rayyan.ai/. 10.1186/s13643-016-0384-4 PMC5139140 27919275

[33] P. F. Whiting , A. W. S. Rutjes , M. E. Westwood , et al., “QUADAS‐2: A Revised Tool for the Quality Assessment of Diagnostic Accuracy Studies,” Annals of Internal Medicine 155, no. 8 (2011): 529–536. 10.7326/0003-4819-155-8-201110180-00009 22007046

[34] J. L. Aguayo‐Albasini , B. Flores‐Pastor , and V. Soria‐Aledo , “Sistema GRADE: Clasificación De La Calidad De La Evidencia y Graduación De La Fuerza De La Recomendación,” Cirugía Española 92, no. 2 (2014): 82–88. 10.1016/j.ciresp.2013.08.002 24361098

[35] J. Lee , K. W. Kim , S. H. Choi , J. Huh , and S. H. Park , “Systematic Review and Meta‐Analysis of Studies Evaluating Diagnostic Test Accuracy: A Practical Review for Clinical Researchers‐Part II. Statistical Methods of Meta‐Analysis,” Korean Journal of Radiology 16, no. 6 (November/December 2015): 1188, 10.3348/kjr.2015.16.6.1189. PMC4644739 26576107

[36] A. Tancin Lambert , B. Ratajczak‐Tretel , R. Al‐Ani , et al., “Biomarkers Predictive of Atrial Fibrillation in Patients With Cryptogenic Stroke: Insights From the Nordic Atrial Fibrillation and Stroke (NOR‐FIB) Study,” European Journal of Neurology 30, no. 5 (2023): 1352–1363, 10.1111/ene.15746. 36786305

[37] A. C. Fonseca , D. Brito , T. P. E Melo , et al., “N‐Terminal Pro‐Brain Natriuretic Peptide Shows Diagnostic Accuracy for Detecting Atrial Fibrillation in Cryptogenic Stroke Patients,” International Journal of Stroke 9, no. 4 (2014): 419–425, 10.1111/ijs.12126. 23981586

[38] J. Pagola , J. Juega , J. Francisco‐Pascual , et al., “Predicting Atrial Fibrillation With High Risk of Embolization With Atrial Strain and NT‐proBNP,” Translational Stroke Research 12, no. 5 (2021): 735–741, 10.1007/s12975-020-00873-2. 33184686

[39] T. Moriyama , K. Todo , H. Yamagami , et al., “Relationship Between Initial B‐Type Natriuretic Peptide Levels and Detection of Atrial Fibrillation With an Insertable Cardiac Monitor in Cryptogenic Stroke: CRYPTON‐ICM Registry,” Frontiers in Neurology 15 (2024): 1436062, 10.3389/fneur.2024.1436062. PMC11445933 39359870

[40] S. M. Kanyu , M. M. Shah , and M. A. Wani , “Diagnostic Accuracy of N‐Terminal Pro‐Brain Natriuretic Peptide (NT‐proBNP) for Detecting Paroxysmal Atrial Fibrillation (PAF) in Cryptogenic Stroke,” International Journal of Medicine and Public Health 14, no. 4 (2024): 520–523, 10.70034/ijmedph.2024.4.98.

[41] G. Y. H. Lip and J. L. Halperin , “Improving Stroke Risk Stratification in Atrial Fibrillation,” American Journal of Medicine 123, no. 6 (June 2010): 484–488, 10.1016/j.amjmed.2009.12.013. 20569748

[42] T. Sanna , H. C. Diener , R. S. Passman , et al., “Cryptogenic Stroke and Underlying Atrial Fibrillation,” New England Journal of Medicine 370, no. 26 (2014): 2478–2486, 10.1056/NEJMoa1313600. 24963567

[43] D. J. Gladstone , M. Spring , P. Dorian , et al., “Atrial Fibrillation in Patients With Cryptogenic Stroke,” New England Journal of Medicine 370, no. 26 (2014): 2467–2477, 10.1056/NEJMoa1311376. 24963566

[44] H. Kamel , M. S. Elkind , R. A. Kronmal , et al., “Atrial Cardiopathy Biomarkers and Atrial Fibrillation in the ARCADIA Trial,” European Stroke Journal 10, no. 2 (2025): 495–501, 10.1177/23969873241276358. PMC11569579 39212178

[45] H. C. Diener , R. L. Sacco , J. D. Easton , et al., “Dabigatran for Prevention of Stroke After Embolic Stroke of Undetermined Source,” New England Journal of Medicine 380, no. 20 (2019): 1906–1917, 10.1056/NEJMoa1813959. 31091372

[46] R. G. Hart , M. Sharma , H. Mundl , et al., “Rivaroxaban for Stroke Prevention After Embolic Stroke of Undetermined Source,” New England Journal of Medicine 378, no. 23 (2018): 2191–2201, 10.1056/NEJMoa1802686. 29766772

[47] D. Šaňák , M. Hutyra , M. Král , et al., “Atrial Fibrillation in Young Ischemic Stroke Patients: An Underestimated Cause?,” European Neurology 73, no. 4 (2015): 158–163, 10.1159/000369793. 25573455

[48] M. Kneihsl , E. Bisping , D. Scherr , et al., “Predicting Atrial Fibrillation After Cryptogenic Stroke via a Clinical Risk Score: A Prospective Observational Study,” European Journal of Neurology 29, no. 1 (2022): 149–157, 10.1111/ene.15102. PMC9292187 34519135

[49] A. M. Louka , S. Nagraj , A. T. Adamou , et al., “Risk Stratification Tools to Guide a Personalized Approach for Cardiac Monitoring in Embolic Stroke of Undetermined Source,” Journal of the American Heart Association 12, no. 18 (2023): e030479, 10.1161/JAHA.123.030479. PMC10547268 37681521

