# Supplementary material for: Natriuretic Peptides as Adjunctive Biomarkers for the Detection of Paroxysmal Atrial Fibrillation in Patients With Cryptogenic Stroke: Systematic Review and Meta‐Analysis
Source: Clin Cardiol. 2026 Nov 5;49(11):e70501. doi: 10.1002/clc.70501 (PMC13739245; doi:10.1002/clc.70501)
Supplement: Supplementary file 1 [file CLC-49-e70501-s001.docx]

**Supplementary Table 1. Detailed search strategy**

**PUBMED (MeSH)**

| PICO question | MeSH terms and keywords |
| --- | --- |
| P (Population) | "Stroke"[MeSH] OR "Ischemic Stroke"[MeSH] OR "Stroke, Embolic"[MeSH] OR "Brain Ischemia"[MeSH] OR "cryptogenic stroke"[tiab] OR "embolic stroke of undetermined source"[tiab] OR ESUS[tiab] OR "ischemic stroke"[tiab] OR stroke[tiab]. |
| I (Intervention / index test) | "Natriuretic Peptides"[MeSH] OR "Natriuretic Peptide, Brain"[MeSH] OR "NT-proBNP"[tiab] OR BNP[tiab] OR "brain natriuretic peptide"[tiab] OR "MR-proANP"[tiab] OR "atrial natriuretic peptide"[tiab] OR "atrial natriuretic peptide"[tiab]. |
| C (Comparator / reference test) | "Electrocardiography, Ambulatory"[MeSH] OR "Telemetry"[MeSH] OR "Event Recorder, Cardiac"[MeSH] OR Holter[tiab] OR "ambulatory ECG"[tiab] OR "ambulatory electrocardiography"[tiab] OR "implantable loop recorder"[tiab] OR "insertable cardiac monitor"[tiab] OR "event recorder"[tiab] OR telemetry [tiab]. |
| OR (Outcome / diagnostic outcome) | "Sensitivity and Specificity" [MeSH] OR "Predictive Value of Tests" [MeSH] OR "ROC Curve" [MeSH] OR sensitivity [tiab] OR specificity [tiab] OR "predictive value" [tiab] OR "diagnostic accuracy" [tiab] OR ROC [tiab] OR "area under curve" [tiab] OR AUC [tiab] OR AUC [tiab]. |

**EMBASE (Emtree)**

| PICO Question | Emtree terms |
| --- | --- |
| P - Population | 'ischemic stroke'/exp OR 'embolic stroke'/exp OR 'brain ischemia'/exp OR 'cryptogenic stroke':ti,ab,kw OR 'embolic stroke of undetermined source':ti,ab,kw OR esus:ti,ab,kw OR 'ischemic stroke':ti,ab,kw OR stroke:ti,ab,kw |
| I - Intervention | 'natriuretic peptide'/exp OR 'brain natriuretic peptide'/exp OR 'natriuretic peptide b-type'/exp OR 'nt-probnp'/exp OR 'atrial natriuretic peptide'/exp OR 'mr-proanp':ti,ab,kw OR bnp:ti,ab,kw OR nt-probnp:ti,ab,kw OR 'brain natriuretic peptide':ti,ab,kw |
| C - Comparator | 'ambulatory electrocardiography'/exp OR 'implantable loop recorder'/exp OR holter:ti,ab,kw OR 'ambulatory ecg':ti,ab,kw OR 'insertable cardiac monitor':ti,ab,kw OR 'event recorder':ti,ab,kw OR telemetry:ti,ab,kw |
| OR - Unlink | 'diagnostic accuracy'/exp OR 'sensitivity and specificity'/exp OR 'roc curve'/exp OR sensitivity:ti,ab,kw OR specificity:ti,ab,kw OR 'predictive value':ti,ab,kw OR auc:ti,ab,kw OR 'predictive value':ti,ab,kw OR auc:ti,ab |

**Cochrane**

| PICO Question | Cochrane Terms CENTRAL |
| --- | --- |
| P - Population | [mh "Ischemic Stroke"] OR [mh "Stroke, Embolic"] OR [mh "Brain Ischemia"] OR "cryptogenic stroke":ti,ab,kw OR "embolic stroke of undetermined source":ti,ab,kw OR esus:ti,ab,kw OR "ischemic stroke":ti,ab,kw OR stroke:ti,ab,kw |
| I - Intervention | [mh "Natriuretic Peptides"] OR [mh "Natriuretic Peptide, Brain"] OR [mh "Natriuretic Peptide, B-Type"] OR [mh "NT-proBNP"] OR [mh "Atrial Natriuretic Peptide"] OR "mr-proanp":[ti,ab,kw OR bnp:ti,ab,kw OR nt-probnp:ti,ab,kw OR "brain natriuretic peptide":ti,ab,kw. |
| C - Comparator | [mh "Electrocardiography, Ambulatory"] OR [mh "Telemetry"] OR [mh "Event Recorder, Cardiac"] OR holter:ti,ab,kw OR "ambulatory ecg":ti,ab,kw OR "ambulatory electrocardiography":[ti,ab,kw OR "implantable loop recorder":ti,ab,kw OR "insertable cardiac monitor":ti,ab,kw OR "event recorder":ti,ab,kw OR telemetry:ti,ab,kw |
| O - Unlink | [mh "Sensitivity and Specificity"] OR [mh "Predictive Value of Tests"] OR [mh "ROC Curve"] OR sensitivity:ti,ab,kw OR specificity:ti,ab,kw OR "predictive value":ti,ab,kw OR "diagnostic accuracy":ti,ab,kw OR auc:ti,ab,kw OR roc:ti,ab,kw |

**Scopus**

| PICO | Scopus strategy (TITLE-ABS-KEY) |
| --- | --- |
| P - Population | TITLE-ABS-KEY ( "ischemic stroke" OR "embolic stroke" OR "cryptogenic stroke" OR "embolic stroke of undetermined source" OR esus OR "brain ischemia" OR stroke ) |
| I - Intervention | TITLE-ABS-KEY ( "natriuretic peptide" OR "brain natriuretic peptide" OR "natriuretic peptide b-type" OR bnp OR "nt-probnp" OR "atrial natriuretic peptide" OR "mr-proanp" ) |
| C - Comparator | TITLE-ABS-KEY ( "implantable loop recorder" OR "insertable cardiac monitor" OR "implantable cardiac monitor" OR "long-term cardiac monitoring" OR "long-term ecg monitoring" OR "continuous ecg monitoring" OR "continuous electrocardiographic monitoring" OR "mobile cardiac outpatient telemetry" OR mcot OR holter OR "holter monitor" OR "holter monitoring" OR "24-hour holter" OR "48-hour holter" OR "ambulatory ecg" OR "ambulatory electrocardiography" OR "ambulatory ecg monitoring" ) |
| OR - Link C | TITLE-ABS-KEY ( "diagnostic accuracy" OR sensitivity OR specificity OR "predictive value" OR "roc curve" OR roc OR auc ) |

**LILACS**

| PICO | LILACS Strategy (BVS) |
| --- | --- |
| P - Population | ( mh: "Ischemic stroke" OR mh: "embolic stroke" OR mh: "cerebral ischemia" OR tw: "cryptogenic stroke" OR tw: "cryptogenic stroke" OR tw: "cryptogenic stroke" OR tw: "embolic stroke of undetermined source" OR tw:ESUS OR tw: "ischemic stroke" OR tw: "ischemic stroke" OR tw: "brain ischemia" ) |
| I - Intervention | ( mh: "Natriuretic Peptides" OR mh: "Brain Natriuretic Peptide" OR mh: "Natriuretic Peptide Type B" OR tw:BNP OR tw: "NT-proBNP" OR tw: "proBNP" OR tw: "MR-proANP" OR tw: "brain natriuretic peptide" OR tw: "brain natriuretic peptide" OR tw: "brain natriuretic peptide" ) |
| C - Comparator | ( mh: "ambulatory electrocardiography" OR mh:telemetry OR tw: "implantable loop recorder" OR tw: "insertable cardiac monitor" OR tw: "insertable cardiac monitor" OR tw: "long-term cardiac monitoring" OR tw: "continuous ecg monitoring" OR tw:"mobile cardiac outpatient telemetry" OR tw:MCOT OR tw:Holter OR tw: "holter monitor" OR tw: "holter monitoring" OR tw: "24-hour holter" OR tw: "48-hour holter" OR tw: "ambulatory ECG" OR tw: "ambulatory electrocardiography" ) |
| OR - Unlinked | ( mh: "Sensitivity and Specificity" OR mh: "Predictive Value of Tests" OR mh: "ROC curve" OR tw:sensitivity OR tw:specificity OR tw:specificity OR tw: "diagnostic accuracy" OR tw: "predictive value" OR tw:ROC OR tw:AUC OR tw: "area under curve" ) |

**Web Of Science**

| PICO | Web of Science Strategy |
| --- | --- |
| P - Population | TS=("ischemic stroke" OR "embolic stroke" OR "cryptogenic stroke" OR "embolic stroke of undetermined source" OR ESUS OR "brain ischemia" OR stroke) |
| I - Intervention | TS=("natriuretic peptide" OR "brain natriuretic peptide" OR "natriuretic peptide b-type" OR BNP OR "NT-proBNP" OR "atrial natriuretic peptide" OR "MR-proANP") |
| C - Comparator | TS=("implantable loop recorder" OR "insertable cardiac monitor" OR "implantable cardiac monitor" OR "long-term cardiac monitoring" OR "long-term ECG monitoring" OR "continuous ECG monitoring" OR "continuous electrocardiographic monitoring" OR "mobile cardiac outpatient telemetry" OR MCOT OR Holter OR "holter monitor" OR "holter monitoring" OR "24-hour holter" OR "48-hour holter" OR "ambulatory ECG" OR "ambulatory electrocardiography" OR "ambulatory ECG monitoring") |
| OR - Unlink | TS=("diagnostic accuracy" OR sensitivity OR specificity OR "predictive value" OR "ROC curve" OR ROC OR AUC) |

**Limitation in search terms:** adults, studies in English and Spanish.

**Supplementary Table S2. Definitions and adjudication of atrial fibrillation across included studies**

| **Study** | **Rhythm monitoring and follow-up** | **AF/PAF definition and minimum episode duration** | **Adjudication process** | **Handling of AHRE or device-detected episodes** |
| --- | --- | --- | --- | --- |
| Rodríguez-Yáñez et al. 2013 (28) | Clinical follow-up by a neurologist at 3 and 6 months and subsequently by a primary care physician for 2 years | New AF diagnosed during follow-up; specific electrocardiographic criteria and minimum episode duration were not reported | Not reported | Not applicable; no ICM was used |
| Fonseca et al. 2014 (37) | 24-h Holter within the first week and at 3 and 6 months when previous monitoring was unremarkable | Dysrhythmia lasting ≥30 continuous seconds, with no detectable P waves and no alternative diagnosis | Reader or formal adjudication process not reported | Not applicable; surface ECG/Holter monitoring |
| Kneihsl et al. 2019 (27) | ≥72 h of inpatient monitoring, including ≥48 h of continuous stroke-unit ECG, 24-h Holter, and regular pulse controls | Arrhythmia lasting >30 continuous seconds, with no detectable P waves and no alternative diagnosis | Reader or formal adjudication process not reported | Not applicable; surface ECG/Holter monitoring |
| Pagola et al. 2021 (38) | 28-day wearable Holter monitoring using the Nuubo™ system | Irregular ECG rhythm lasting >120 s was classified as possible AF. Patients were subsequently categorized according to the longest episode as no AF, AF <5 h, or AF >5 h (HpAF) | Episodes were verified by an expert cardiologist blinded to clinical data | Not applicable; algorithm-generated possible AF episodes required cardiologist verification |
| Palà et al. 2021 (26) | 28-day wearable Holter monitoring using the Nuubo™ system | Irregular ECG rhythm lasting >120 s was classified as possible AF | Holter recordings were reviewed and AF episodes confirmed by an expert cardiologist blinded to patient characteristics and clinical data | Not applicable; algorithm-generated possible AF episodes required cardiologist verification |
| Tancin Lambert et al. 2023 (36) | Reveal LINQ® ICM monitoring for 12 months | Minimum episode duration of 2 min, based on the device detection algorithm. The study reported detection of AF or atrial flutter | Transmitted ECG recordings were reviewed weekly by a group of two neurologists and two cardiologists | AHREs were not reported as a separate outcome. Verified episodes meeting the ≥2-min criterion were treated as AF/atrial flutter outcomes |
| Kanyu et al. 2024 (40) | ≥72 h of in-hospital cardiac rhythm monitoring or Holter monitoring | PAF detected during monitoring; specific electrocardiographic criteria and minimum episode duration were not reported | Not reported | Not applicable; no ICM was used |
| Moriyama et al. 2024 (39) | ICM monitoring using Reveal LINQ®, Confirm Rx®, or BioMonitor 2-AF®; median follow-up of 579 days | Irregular heart rate lasting ≥2 min | Device alerts were reviewed and judged by the study physician through a remote monitoring system | AHREs were not reported as a separate outcome. Physician-reviewed episodes meeting the ≥2-min criterion were classified as AF |

*AF, atrial fibrillation; AHRE, atrial high-rate episode; ECG, electrocardiogram; HpAF, paroxysmal atrial fibrillation with high embolic risk; ICM, insertable cardiac monitor; PAF, paroxysmal atrial fibrillation.*

**Supplementary Figure S1.** Deeks´ funnel plot for assessing publication bias among the included studies for NT-proBNP.

The X-axis represents the inverse root of the effective sample size, and the Y-axis displays the log of the diagnostic odds ratio. The symmetry of the plot is used to evaluate the presence of bias. A significant p-value (p = 0.07) suggests risk of publication bias. This finding should be interpreted cautiously given the small number of individual included studies (n=8)

**Supplementary Figure S2. Forest plot of sensitivity and specificity of natriuretic peptides for screening paroxysmal atrial fibrillation stratified by manufacturer's platform.**

**Supplementary Figure S3. ROC curves for NT-proBNP and BNP according to age** a) ≤65 years. b) ≥70 years. c) ≥75 years.

a) b)

c)
